# Tissue-Specific Tumour Suppressor and Oncogenic Activities of the Polycomb-like Protein MTF2

**DOI:** 10.3390/genes14101879

**Published:** 2023-09-27

**Authors:** Mzwanele Ngubo, Fereshteh Moradi, Caryn Y. Ito, William L. Stanford

**Affiliations:** 1 The Sprott Centre for Stem Cell Research, Ottawa Hospital Research Institute, Ottawa, ON K1H 8L6, Canada; 2 Ottawa Institute of Systems Biology, Ottawa, ON K1H 8M5, Canada; 3 Department of Cellular and Molecular Medicine, University of Ottawa, Ottawa, ON K1N 6N5, Canada; 4 Department of Biochemistry, Microbiology and Immunology, University of Ottawa, Ottawa, ON K1H 8M5, Canada

**Keywords:** Polycomb repressive complex 2, Polycomb-like, PHF1, MTF2, PHF19, tumour suppressor, oncogene, cancer, development, epigenetics

## Abstract

The Polycomb repressive complex 2 (PRC2) is a conserved chromatin-remodelling complex that catalyses the trimethylation of histone H3 lysine 27 (H3K27me3), a mark associated with gene silencing. PRC2 regulates chromatin structure and gene expression during organismal and tissue development and tissue homeostasis in the adult. PRC2 core subunits are associated with various accessory proteins that modulate its function and recruitment to target genes. The multimeric composition of accessory proteins results in two distinct variant complexes of PRC2, PRC2.1 and PRC2.2. Metal response element-binding transcription factor 2 (MTF2) is one of the Polycomb-like proteins (PCLs) that forms the PRC2.1 complex. MTF2 is highly conserved, and as an accessory subunit of PRC2, it has important roles in embryonic stem cell self-renewal and differentiation, development, and cancer progression. Here, we review the impact of MTF2 in PRC2 complex assembly, catalytic activity, and spatiotemporal function. The emerging paradoxical evidence suggesting that MTF2 has divergent roles as either a tumour suppressor or an oncogene in different tissues merits further investigations. Altogether, our review illuminates the context-dependent roles of MTF2 in Polycomb group (PcG) protein-mediated epigenetic regulation. Its impact on disease paves the way for a deeper understanding of epigenetic regulation and novel therapeutic strategies.

## 1. Introduction

The chromatin state, or epigenome, is dynamically regulated and plays central roles in cell identity and genome stability. Integrating environmental signals with transcriptional networks, activating and repressive epigenomic complexes direct cell fate decisions in developmental programs and tissue homeostasis. As a result, alterations to the epigenome can lead to developmental abnormalities, chronic diseases, and cancer [1,2,3]. Drugs targeting epigenetic regulators are promising therapeutics for a variety of diseases. Polycomb group (PcG) proteins modulate epigenetic states. They were discovered by their repression of homeotic genes [4,5]. Polycomblike (*Pcl*) was identified via a Polycomb mutation enhancer screen in *Drosophila melanogaster* [6]. The Pcl2 protein metal-regulatory transcription factor 2 (MTF2) is one of the three Pcl homologs encoded in vertebrates [7,8,9]. It has been shown to regulate mouse development and the transition of pluripotent stem cells to differentiated progenitors [9,10,11,12]. We recently showed that MTF2 regulates chemotherapeutic response in acute myeloid leukemia (AML), and since the levels of MTF2 and H3K27me3 are correlated with response to induction chemotherapy, they may be useful biomarkers to prospectively identify patients with chemoresistant disease at diagnosis [13,14]. Additional studies have implicated MTF2 in the progression of various cancers, for which it may also serve as a prognostic and therapeutic biomarker [15,16,17,18,19,20,21]. Here, we will review published studies and present data mined from publicly available databases to consider a variety of potential context-specific roles of MTF2 in cancer.

MTF2 function has been ascribed through its association with other PcG proteins. Polycomb genes were initially identified during screening for developmental defects in *Drosophila melanogaster* [22,23]. PcG proteins regulate the transcription of developmental genes involved in a wide range of dynamic biological processes such as differentiation, stem cell plasticity, or cell cycle progression. Furthermore, mutations or dysregulation of PcG proteins have been extensively described in the context of cancer initiation, progression, and metastasis [13,24,25,26]. PcG proteins assemble in large distinctive multimeric protein complexes with different catalytic activities and cellular functions, which are broadly classified as Polycomb repressive complexes 1 and 2 (PRC1 and PRC2), that act synergistically to establish a repressive chromatin state at their target genes [3]. At least six discrete PRC1 complexes have been identified (all sharing a heterodimeric PCGF-RING core) [27]. All PRC1 complexes possess E3 ubiquitin ligase activity that, in mammalian cells, targets Lys118 and Lys119 of histone H2A (H2AK118ub and H2AK119ub) (K117 and K118 in *Drosophila melanogaster*), which is key for the modular interplay of the different Polycomb complexes [28,29,30]. PRC2 in mammals is composed of core constitutive subunits: the embryonic ectoderm development (EED); suppressor of zeste 12 homolog protein (SUZ12); enhancer of zeste homolog 1 (EZH1) or EZH2 [31].

Core subunits associate with equimolar stoichiometries and they bind to a variety of “accessory” proteins that extend and customise the activity of the whole complex. Methyltransferase activity that mono-, di-, or tri-methylates the lysine 27 of the histone H3 (H3K27me1/2/3) is performed by the EZH1 or EZH2 subunit, and SUZ12 functions as a backbone by orchestrating the cofactor interactions and stability of the complex. In PRC2, the stability of the core subunits EED, SUZ12, and EZH1/2 is interdependent, and the steady state of the core complex is also critical for its methyltransferase activity. EED recognises H3K27me3 and allosterically activates EZH1/2 (mainly EZH2), enabling the spreading of H3K27me3. Primarily, EED binds to H3K27me3 through its WD40 domain, causing the SET (Su(var)3–9, Enhancer-of-Zeste and Trithorax) domain of EZH1/2 to be configured into the active conformation, allosterically activating its catalytic activity [32,33].

Mono-methylation of H3K27me2 results in H3K27me3, which is a stable mark, but the importance of H3K27me2 in maintaining gene repression is not well understood [34]. H3K27me2 functions as an intermediary PRC2 product that not only constitutes a substrate for subsequent H3K27me3 formation but may also prevent H3K27 from being acetylated. Acetylated H3K27 is speculated to antagonise PcG-mediated gene silencing and is in abundance in the absence of PRC2 [35]. Unlike H3K27me2/3, H3K27me1 is still detectable in cells carrying non-functional PRC2, and its enrichment correlates with actively transcribed genes [36,37]. Exactly how H3K27me1 is generated is debatable. H3K27me1 may be catalysed by PRC2, and its presence in actively transcribed genes also results from the demethylation of H3K27me2/3 by the demethylases lysine demethylase 6B (KDM6B) or histone demethylase (UTX).

By itself, the PRC2 core complex lacks the capability to be recruited to target genes and has a significantly reduced capacity for binding chromatin. Several accessory subunits have now been identified to regulate PRC2 function. Recent comprehensive proteomic analyses have demonstrated that they form two main PRC2 subtypes, namely, PRC2.1 and PRC2.2, which are characterised by the association with specific accessory proteins and are differentially regulated, demonstrate different interactions and are recruited to DNA by independent mechanisms [38]. Although the general repressive role of PRC2 is preserved throughout evolution, its functionality has diversified remarkably, and PRC2.1 appears to be more heterogeneous than PRC2.2 [39].

## 2. PRC2 Variant Complexes

**Figure 1:**
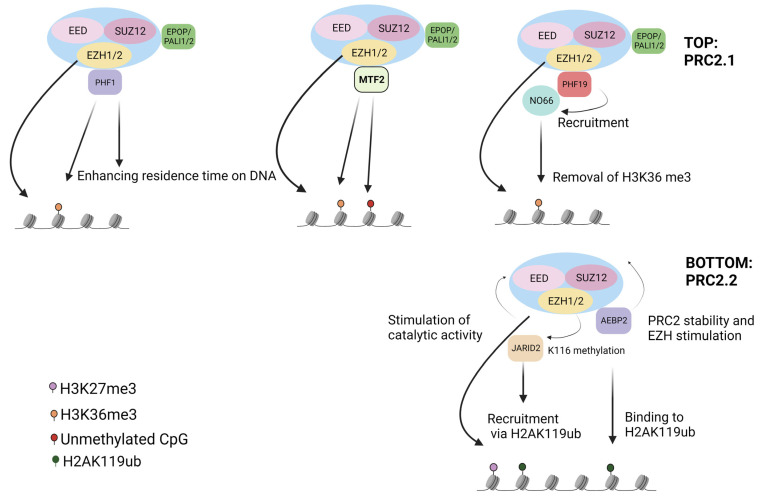
**An overview of PRC2 variants.** Top (PRC2.1): PHF1 can associate with H3K36me3, recruit PRC2.1 to genomic regions containing high H3K36me3 levels, and also extend the residence time of PRC2.1 on chromatin. MTF2 recruits PRC2.1 to unmethylated CpG islands and also binds to H3K36me3 loci. PHF19 associates with H3K36me3 and recruits NO66, which demethylates H3K36me3, facilitating H3K27me3 deposition. EPOP has inhibitory effects on the complex by association with EloB/C to maintain low levels of transcription. PALI1/2 boosts the catalytic activity of EHZ1/2. Therefore, EPOP and PALI1/2 fine-tune the activity of PRC2 at target genes. Bottom (PRC2.2): JARID2 can recruit PRC2.2 by binding H2AK119ub and augment the catalytic activity of EZH1/2. JARID2 can also be methylated at K116 by EZH2, which mainly functions as a binding scaffold for EED, culminating in the stimulation of EZH2 catalytic activity. AEBP2 is a stabilising c-factor of PRC2.2 and can increase the catalytic activity of EZH1/2.

PRC2.1 contains one of the three paralogous PCL proteins, PCL1/2/3, also referred to as PHF1, MTF2, and PHF19, respectively (Figure 1, Top). Emerging results showed that mammalian PRC2.1 not only contains a PCL protein but also incorporates additional subunits, for which no homologs are found in *Drosophila melanogaster*. PRC2-associated LCOR isoform 1/2 (PALI1/2, also referred to as C10ORF12) or Elongin B/C and PRC2- associated protein (EPOP, also known as C17ORF96) have been reported to associate with PRC2.1 complex. EPOP has demonstrated inhibitory effects on PRC2 chromatin binding, thereby maintaining expression of lowly transcribed genes [40,41]. The protein SKIDA1 (C10ORF140), which shares the PRC2-binding domain with EPOP, was also found to be associated with PRC2.1; no homologs of these non-PCL PRC2-associated proteins have been identified in *Drosophila melanogaster.* There is evidence showing that in PRC2.1, EPOP and PALI1/2 compete for binding, although its functional implication is unknown [42]. PRC2.1 contains at least one of two methyltransferases (EZH1 or EZH2) and one of the three PCL (PHF1, MTF2, or PHF19), making six possible combinations. The addition of EPOP, SKIDA1, PALI1, or PALI2 accessory proteins culminates in twenty-four further putative PRC2.1 complexes. Notably, the significance of forming one combination versus the other possibilities is undetermined. Additionally, structural features of the PRC2 holocomplex can restrict this number of combinations, as discussed in this review [43].

The other variant, PRC2.2, is comprised of Jumonji and AT-rich interaction domain containing 2 (JARID2) and adipocyte enhancer-binding protein 2 (AEBP2) subunits in equal stoichiometry. JARID2 is suggested to be integral for PRC2 targeting at its loci, possibly through its DNA-binding domain (Figure 1, bottom). These accessory subunits—JARID2 and AEBP2—are also important for PRC2 recruitment, via recognition of the H2AK119ub mark, as well as for the deposition of H3K27me3 at specific PcG targets. Though the accessory subunits of PRC2 have been identified, their precise roles are not fully discerned. This can be partially explained by the compensatory mechanisms employed by both variants, as elegantly demonstrated by combined ablation of JARID2 and all three Pcl genes in mouse embryonic stem cells (ESCs) [44,45]. Additionally, in the absence of AEBP2, JARID2 and MTF2 were found to associate in the same complex [46], indicating that the distinction between PRC2.1 and PRC2.2 is not firm, and that hybrid PRC2 complexes can form when PRC2.1 and PRC2.2 bind non-overlapping interaction loci [47].

### PRC2.1 Gene Recruitment

PRC2 core subunits lack sequence-specific DNA-binding domains that would direct recruitment to their target genes. Thus, Polycomb-mediated gene regulation relies on components that direct their recruitment to specific chromatin domains. In a classical model, described in *Drosophila melanogaster*, PRC2 is first recruited to cis-regulatory sequences called Polycomb Response Elements (PREs) via consensus motifs for sequence-specific DNA-binding proteins that might interact with PRC2 subunits. Though the existence of mammalian PREs is highly controversial, the analysis of PRC2 genome binding identified the enrichment of CpG islands characterised by low levels of DNA methylation that are likely to serve as the equivalent of PREs in mammals. The PCL proteins PHF1, MTF2, or PHF19 preferentially bind unmethylated CpG-containing DNA sequences, promoting PRC2 binding to CpG islands and stabilising the dimerisation of PRC2 [41,42,46,48]. PRC2 can also be modulated by non-coding RNAs (ncRNAs), including long non-coding RNAs (lncRNAs) [49,50,51]. These ncRNAs vary in size, ranging from small ncRNAs of tens of nucleotides to long ncRNAs spanning hundreds of kilobases. Some long ncRNAs can fold into secondary structures and establish specific DNA interactions. These discoveries have rendered the interaction between PcGs and lncRNAs a compelling mechanism for the recruitment of PcGs to specific genomic sites [52]. The chromatin landscape can be a recruitment determinant factor. For example, Trithorax group proteins oppose the Polycomb-mediated gene silencing by modifying chromatin with active histone marks such as H3K4me1/2/3 [5]. The simultaneous occurrence of H3K4me3 (associated with TrxG) and H3K27me3 modification patterns (related to PcG) is termed as a bivalent domain, which results in genes that are poised for activation [53]. Collectively, the exact molecular mechanisms undertaken by PcG complexes to specifically target the genome remain an area of interest, with important ramifications for understanding the specificity of target genes. 

**Figure 2:**
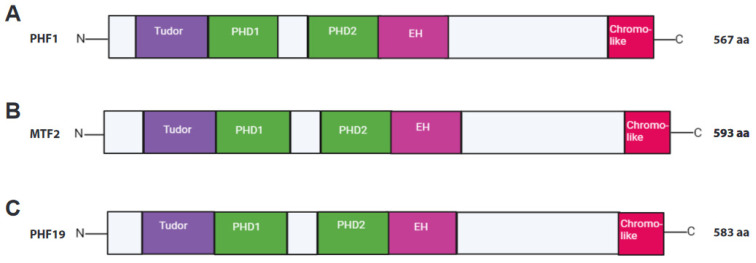
**Overview of domain structure of PCLs.** PCL protein domains are shown from N-C terminus, left to right, in: (**A**) PHF1 (PCL1), (**B**) MTF2 (PCL2), and (**C**) PHF19 (PCL3).

Structurally, the PCL proteins possess five modular domains, namely, one Tudor domain (exclusive to PCLs among all other PRC2-interacting proteins), two plant homeodomain (PHD) zinc fingers, an extended homologous (EH) region, and a chromo-like domain located at the C-terminus (illustrated in Figure 2). Li et al. demonstrated that the EH regions of PCL proteins fold into a winged-helix structure, which binds to the unmethylated CpG loci. However, the mode of binding differs substantially from that displayed by proteins with a canonical winged-helix DNA recognition motif [54]. In addition to binding to DNA and extending the residence time of PRC2 on chromatin, Phf1 also augments the catalytic activity of PRC2, mainly by its association with H3K36me3 (which is known to inhibit the enzymatic activity of the PRC2 complex). Like Phf1, Phf19 can modulate the recruitment and catalytic function of PRC2. In mouse ESCs, Phf1 and Phf19 have approximately tenfold lower expression levels relative to Mtf2. Indeed, the number of Mtf2-containing PRC2.1 significantly outnumbers the PRC2.1 with either Phf1 or Phf19 [55,56,57]. Unlike Mtf2, the loss of Phf1 and Phf19 did not show a similar level of marked reduction in PRC2 recruitment [44,58]. This may be because these proteins are expressed at low levels compared to Mtf2 [57].

## 3. MTF2 Structural Function

The MTF2 locus (human chromosome 1, mouse chromosome 5) encodes four alternatively spliced isoforms in human and eight in mice (RefSeq, GRCh38 (human): NM_001164391.2, NM_001164392.2, NM_001164393.2, NM_007358.4; RefSeq, GRCm39 (mouse): NM_013827.3, NM_001253877.1, NM_001253878.1, NM_001253879.1, NM_001253880.1, NM_001359089.1, NM_001359088.1, NR_152817.1). Though the specific roles have not been identified, an appealing hypothesis is that each isoform of MTF2 has specific protein- or DNA-binding targets. Each isoform has yet to be studied independently in detail, but based on its lack of the Tudor domain, it is likely that the shortest isoform plays a distinct role or has a unique set of targets. Both human and murine truncated transcripts lack a Tudor domain [7,59]. The Tudor domain is not found in other PRC2-interacting proteins that may confer a unique role to PCL proteins and PRC2.1.

The Tudor domain belongs to the ‘‘Royal family’’ of protein–protein interacting domains [60], which are implicated in diverse epigenetic functions, including methylation-dependent chromatin remodelling, histone binding, pre-RNA processing, RNA silencing, transposon silencing, and microRNA-mediated gene silencing [61,62]. In PCL proteins, the Tudor domain forms an aromatic cage that can link PRC2 to chromatin by recognising the active H3K36me3 histone mark [59,63,64,65,66,67]. Hence, PCL proteins serve as both bridges and targeting mechanisms, facilitating intrusion by PRC2.1 into active chromatin loci. At the same time, the relevance of the H3K36me3-binding function of this region remains less obvious. Speculatively, both the targeting and spreading of PRC2 into active chromatin regions marked by H3K36me3 could be essential to maintain optimal repression of “poised” developmental genes.

In addition to DNA binding, PHD domains have also been demonstrated to bind RNA, proteins, and lipids [68,69]. Deletion analysis and site-directed mutagenesis demonstrated that both PHD domains are important in mediating the interaction of PCL proteins with EZH1/2 [70]. These domains are characterised by a highly conserved Cys4-His-Cys3 motif identified in several proteins involved in modulating transcription and protein–DNA and protein–protein interactions. Structural studies have shown that the PRC2 core component EZH1/2 is shown to be positioned in three-dimensional space in proximity to the Tudor domain (Lys68) and the first PHD domain (Lys161). DNA binding primarily relies on the EH region, which folds into a winged-helix structure [54,71]. Moreover, the PHD finger 1 of PHF1 has been proposed to interact with symmetrically dimethylated arginine at position 3 of histone 4 (H4R3me2s) to harmonise H4R3me2s and H2AK119ub1 modifications for transcriptional silencing [72]. Whether the PHD domains of MTF2 interact with various other chromatin modifications has not yet been determined.

Crystallisation of MTF2 with a short DNA sequence suggests that the W1 loop of the EH domain enters into the major groove and directly binds the CpG of the DNA bait [54]. Additionally, the EH domain has been shown to augment PRC2 binding to chromatin through a direct interaction with the linker DNA and to facilitate PRC2 binding to CpG islands [54,71,73]. The PCL proteins do not behave identically in their ability to interact with DNA. Whereas MTF2 and PHF1 avidly bind to DNA, the DNA-binding potential of PHF19 is much weaker [54]. Furthermore, MTF2 has been shown to bind to a subset of unmethylated CpG-dense DNA loci directly and selectively, with preferential binding to loci with underwound helixes compared to the canonical B-DNA structure, thereby narrowing potential target regions [54,73]. In summary, these findings illustrate that the DNA-binding function constitutes a major feature of the PCL proteins by which they recruit PRC2.1 to the genomic targets, and MTF2 has a distinct preference to bind to a decreased helix twist.

## 4. Regulation of ESC Fate by MTF2

Following uterine implantation, the cells of the blastocyst inner cell mass (ICM) initiate gastrulation and differentiate into the entire adult organism. ESCs arise from cultured ICM explants. They can be cultured as pluripotent stem cells or allowed to differentiate in vitro or in vivo, as they are governed by the same signalling and gene regulatory networks (GRNs) of the developing embryo [74]. As non-transformed pluripotent cells that can be propagated or differentiated into specific tissues, ESCs are an ideal model for molecular, developmental, and disease modelling studies. As such, the role of Polycomb complexes in the regulation of stem cell fate and developmental programs has been extensively studied in ESCs.

MTF2 was identified as a regulator of pluripotent stem cell fate via dynamic GRN modelling analysis [75]. It has been shown to be a functional component of PRC2 and to be involved in gene recruitment of PRC2 in ESCs regulating pluripotency [10,11,76,77]. *Mtf2* is highly expressed in mouse ESCs, and its promoter is bound by Oct4 and Nanog, implicating that it is subject to regulation by pluripotency transcription factors. The disruption of Mtf2 has no effect on the expression of core PRC2 complex subunits [10,76], reaffirming its function as a PRC2 accessory protein in ESCs. Mtf2 is a critical node regulating ESC self-renewal and commitment GRNs [10]. Knockdown of Mtf2 results in upregulation of key transcription factor nodes of the extended pluripotency network, such as Tbx3, Klf4, and Foxd3, which are known to positively regulate self-renewal through the stabilisation of the Oct4/Sox2/Nanog pluripotency network [10]. More recently, Loh et al. [11] demonstrated that Mtf2-containing PRC2.1 plays a determinant role in balancing poised lineage-specific gene activation, the exit of pluripotency and lineage choice. Single-cell transcriptomic analyses of mouse embryoid bodies yielded from Jarid2 and Mtf2 ESC mutants revealed that Mtf2 mutant cells differentiated faster into all germ layers, whereas the Jarid2 mutants are hindered and principally give rise to early differentiating precursors with a slower downregulation of pluripotency genes. Notably, loss of Mtf2 resulted in enhanced key lineage-specific transcription factors and H3K4me3 modifications with concomitant decreased H3K27me3. In contrast, Jarid2 deficiency led to subtle de-repression of a partially distinct and functionally more selective set of genes [11]. These results show that Mtf2 and Jarid2 differentially regulate poised lineage-specific gene activation and the exit of the pluripotent state.

Additionally, Mtf2 and Jarid2 were shown to be required for normal differentiation of ESCs to neural progenitor cells (NPCs) by affecting distinct sets of Polycomb target genes [78]. Acute depletion of either Mtf2 or Jarid2 resulted in the deregulation of hundreds of genes during the differentiation of ESCs to NPCs [78], implying that both Polycomb accessory factors have distinct roles in gene regulation during this cell identity transition. Additionally, it was shown that Jarid2, Mtf2 and Epop are highly expressed in mouse ESCs compared to differentiated cells, and crucially, a reduction in Jarid2, Mtf2, or Epop significantly impacts the extent of PRC2-mediated H3K27 methylation and leads to the activation of genes associated with cellular differentiation [79]. Consistent with their role in maintaining pluripotency, the simultaneous expression of Jarid2, Mtf2, and Epop improves epigenetic reprogramming of mouse embryonic fibroblasts into induced pluripotent stem cells. However, individual expression of Jarid2, Mtf2, and Epop does not yield the same effect. Conversely, reprogramming is considerably inhibited when Jarid2, Mtf2, or Epop is knocked down or knocked out [79]. Hence, these data confirm the critical, non-redundant roles of PRC2.1 and PRC2.2 accessory proteins in establishing and regulating the pluripotency GRN.

Genes uniquely sensitive to Mtf2 depletion show high CpG densities as well as high levels of H3K27me3 in both ESCs and NPCs, suggesting that Mtf2 is necessary to maintain H3K27me3 at a group of “strong” PRC2 targets that remain repressed during the ESC–NPC transition. However, some PRC2 targets with comparably high CpG densities were not sensitive to Mtf2 depletion [78], implying the presence of other factors contributing to this requirement. Collectively, these observations confirm the important function of Mtf2 in mediating the regulatory role of PRC2 in pluripotency network and differentiation.

## 5. Developmental Roles of MTF2

A study in *Xenopus* provided the first insight into in vivo function of Mtf2 (XPcl2) in gain-of-function studies. Overexpression of Mtf2 (XPcl2) disrupted anterior-posterior patterning of the neural tissues [9]. In the chick, Mtf2 was identified via a subtractive cDNA screen as asymmetrically expressed in Hensen’s node [12]. Ectopic expression of chick Pcl2 in both chick embryos and mouse hindlimb buds resulted in repression of sonic hedgehog and left-right patterning defects [12].

In the mouse, widespread embryonic Mtf2 expression in the gastrulating embryo becomes restricted as organogenesis proceeds [80,81,82]. Complementing the non-mammalian gain-of-function studies, several loss-of-function mutations have been reported in the mouse. The first allele reported, *Mtf2^Gt(XD143)Byg^*, encoded a gene trap insertion upstream of exon 4. Homozygous gene trap mice were viable and demonstrated normal left-right patterning, although a quarter of the *Mtf2^Gt(XD143)Byg^* homozygous mice exhibited skeletal abnormalities commonly associated with homeotic transformation as well as growth retardation. However, thorough evaluation of transcript or protein expression was not performed to determine the nature of the loss-of-function (e.g., hypomorphic or null) mutation [80].

The Koseki lab reported two *Mtf2* alleles, the first being derived from a gene trap in exon 3 (*Mtf2^Gt(U3Betageo)1Ruiz^*) that resulted in a hypomorphic mutation as determined by Western blotting. The authors also used conventional gene targeting of exons 4 and 5 (allele *Mtf2^delta^*), which generated a predominantly truncated transcript lacking the Tudor domain. Homozygous mice from either allele resulted in posterior transformation of the vertebrae and ribs and early postnatal lethality with incomplete penetrance [81]. Strikingly, in situ hybridisation of HOX genes Hoxb4, Hoxb6, Hoxb8, and Hoxd4 showed the anterior boundaries in the developing sclerotome were shifted cranially, confirming a role of Mtf2 in anterior-posterior specification. Genetic interactions between Mtf2, PRC2, and PRC1 complexes were established by intercrossing *Mtf2^delta/delta^* mice with heterozygous (Suz12) or homozygous mice (Pcgf2 or Phc2), which demonstrated more severe homeotic transformations in the compound mutant mice [81].

Finally, we generated gene-targeted *Mtf2* knockout mice by deleting exons 4–6 [82]. These targeted mice resulted in the loss of detectable protein by Western blotting and embryonic lethality by embryonic day (e) 15.5. Like the other *Mtf2* mutant alleles, homozygous *Mtf^−/−^* embryos displayed skeletal alterations, including fusion of the vertebrae and ectopic ribs. As detailed in the next section, *Mtf2^−/−^* embryos died due to severe anaemia [82].

## 6. MTF2 Is Essential for Definitive Erythropoiesis and Regulating Wnt Signalling in Erythroid Cells

Mtf2^−/−^ embryos also displayed growth defects and haemorrhaging, although these phenotypes may be secondary effects of anaemia. Flow cytometric analyses revealed that Mtf2 expression is elevated in long-term and short-term hematopoietic stem cells (HSCs), progenitor cells, and throughout various stages of erythroblast development (identified by the expression of CD71 and/or Ter119) [82]. During the cell cycle of erythroblasts, the expression of Mtf2 was modulated, with the highest levels observed during the S and G2/M phases. This dynamic expression pattern throughout the cell cycle closely resembled that of the core proteins PRC2 Suz12 and Ezh2. Mtf2^−/−^ embryos contained mainly primitive nucleated erythroblasts, with few mature enucleated red blood cells (RBCs) generated by definitive erythropoiesis. Clonogenic assays of fetal liver cells showed that Mtf2^−/−^ embryos had reduced erythroid progenitors but increased multipotent progenitors, implicating a block in erythroid differentiation. This was confirmed by knockdown of Mtf2 in bone marrow (BM) hematopoietic stem and progenitor cells (HSPCs), which phenocopied the block in erythropoiesis [82]. This contrasts with the cell non-autonomous role of Jarid2 in erythropoiesis [83].

Flow cytometric analyses determined that erythroblast differentiation was blocked at the transition of pro-erythroblast CD71^+^Ter119^−/lo^ to CD71^+^Ter119^+^ erythroblasts. To examine the underlying molecular defect, RNA-seq and H3K27me3 and Mtf2 ChIP-seq were performed on fetal liver-derived (e14.5) cells from these two populations. Mtf2-null cells displayed a marked genome-wide loss of H3K27me3 in approximately 2400 genes compared to WT cells. Nearly half of these genes were also bound by Mtf2 at this stage of development. Notably, there is minimal overlap between genes linked to Mtf2 binding peaks in pro-erythroblasts or erythroblasts and the Mtf2 or other PRC2 binding profiles in ESCs [10,84]. This finding highlights the cell type-specific interactions of Mtf2 with chromatin, emphasising its distinct role in different cellular contexts.

Integration of RNA-seq and ChIP-seq datasets imputed an Mtf2 GRN in erythroblasts. High-confidence predicted network modules included genes (i.e., network nodes) in which Mtf2 binding peaks were identified in the promoter-proximal regions containing non-methylated CpG islands of genes that exhibited reduced H3K27me3 levels at the transcription start site and increased mRNA levels in Mtf2^−/−^ erythroblasts. In addition to the activation of cell proliferation networks, the Mtf2 erythroid GRN predicted that the Wnt/β-catenin signalling is de-repressed in Mtf2^−/−^ erythroblasts. Considering the known role of PRC2 in regulating the Wnt pathway in a variety of tissues [85,86], we tested the role of Wnt signalling in Mtf2-deficient erythroblasts. Mtf2^−/−^ fetal liver progenitors demonstrated increased β-catenin nuclear localisation, consistent with increased Wnt signalling, which was phenocopied by Mtf2 knockdown of adult BM HSPCs. Furthermore, knockdown of Mtf2 in adult BM HSPCs led to the loss of H3K27me3 marks at the promoter region of β-catenin and an increase in its mRNA levels, indicating that Mtf2, along with PRC2, epigenetically suppresses the expression of β-catenin in both adult and fetal liver HSPCs. Finally, small-molecule Wnt inhibitors rescued defective erythropoiesis in Mtf2-deficient BM HSPCs. Collectively, these data confirm that Mtf2-PRC2 functions in a feed-forward network to repress Wnt signalling and allow erythroid differentiation [82].

## 7. The Role of MTF2 in Acute Myeloid Leukaemia (AML)

Changes in H3K27me3 levels and distribution are a hallmark of transformation in many malignancies [87,88,89,90]. The phenotypes of increased self-renewal and block in differentiation in Mtf2-deficient ESCs and erythroblasts, respectively, are consistent with AML. Furthermore, probing the Human Protein Atlas [91] demonstrates that MTF2 is highly differentially expressed, with the highest expression in the bone marrow, lymph node, and testis. Therefore, we tested diagnostic AML samples for H3K27me3 and MTF2 expression levels. Initially, we analysed global H3K27me3 levels by flow cytometry in bulk BM cells or various HSPC populations (CD34^+^CD38^−^, CD34^+^CD38^+^, CD34^+^) isolated from diagnostic AML BM aspirates from patients who have received standard-of-care induction chemotherapy in a double-blind fashion [13].

Generally, patients were grouped into those with H3K27me3 levels similar to control BM samples and those with substantially decreased levels. Strikingly, the majority of AML patients with decreased H3K27me3 levels in diagnostic BM cells failed to respond to treatment. Patients who are non-responsive to therapy are known as refractory. Depending on age, they can represent more than 30% of patients with AML. Notably, low H3K27me3 levels within the enriched leukemic stem cell subpopulation (CD34^+^CD38^−^) [92] predicted chemotherapy resistance (refractoriness) and poor patient survival [92]. Additionally, H3K27me3 levels within patient CD34^+^CD38^−^ cells significantly correlated with *MTF2* mRNA expression, whereas other PRC2 members demonstrated weaker correlation with H3K27me3 levels. These findings support the results from Rothberg et al., which demonstrate that MTF2 has essential functions in the hematopoietic system [82]. Additionally, analysis of a cohort of more than 160 AML patients treated by induction chemotherapy in The Cancer Genome Atlas (TCGA) showed that low *MTF2* transcript levels in bulk BM RNA correlated with lower survival [13]. 

MTF2 is rarely mutated in AML and targeted sequencing of the MTF2 locus in a cohort of local AML patients confirmed the lack of mutations. In contrast, we determined that at least one of the two CpG islands was hypermethylated in MTF2-reduced AML samples, whereas neither healthy BM nor AML samples with normal (basal) levels of H3K27me3 had methylated CpG islands, confirming that MTF2 deficiency in AML is mainly mediated by epigenetic means [13]. However, the molecular pathways underlying the methylation of *MTF2* CpG islands in AML is unknown. AML is a highly heterogeneous hematopoietic malignancy and sequencing results uncovered epigenetic modifiers as the most frequently mutated class of genes [93]. Thus, a more comprehensive epigenetic landscape associated with refractory AML could elucidate the cause of hypermethylated *MTF2* CpG islands in MTF2-deficient cells. Given that AML has been shown to harbour dynamic clonal evolution at relapse, including the addition of new mutations that could be relevant for relapse pathogenesis [92], evaluating the MTF2 modifications at this stage of disease would be worthwhile to explore.

Primary MTF2-deficient AML cells were resistant to apoptosis upon treatment with induction therapy drugs cytarabine or daunorubicin, and rescuing MTF2 expression by lentiviral expression increased H3K27me3 and promoted apoptosis following induction drug treatment. Notably, knockdown of MTF2 in control human HSPCs induced resistance to induction chemotherapy drugs cytarabine or daunorubicin, demonstrating that loss of MTF2 expression is sufficient to induce chemotherapy refractoriness [13]. 

H3K27me3 ChIP-seq analysis of HSPCs, MTF2 knockdown HSPCs, refractory AML, and chemosensitive AML demonstrated that loss of MTF2 leads to an epigenetic state similar to refractory AML. Integration of ChIP-seq data and RNA-seq of control and MTF2 knockdown HSPCs identified dysregulation of numerous DNA damage response (DDR), proliferation, and cell survival/apoptosis GRNs. Of particular interest, the PI3K-AKT pathway and its downstream target MDM2, an E3 ubiquitin ligase that negatively regulates p53, were demonstrated to be de-repressed in MTF2-deficient cells. Under normal circumstances, activation of the p53 pathway blocks the proliferation of cells harbouring damaged DNA [94]. In AML, loss-of-function mutations in the gene encoding p53 (*TP53*) are associated with dire outcomes such as chemoresistance, refractory disease, adverse risk group, and poor survival [93]. Cell and molecular analyses validated that MDM2 is a direct target of MTF2/PRC2 and is upregulated in MTF2-deficient refractory AML, leading to the repression of p53-mediated apoptosis in response to treatment [13]. Inducing MTF2 expression in refractory AML cells or small-molecule MDM2 inhibitors sensitised AML cells to induction chemotherapy drugs, inducing cell death. Moreover, treatment of refractory AML patient-derived xenograft mice using an MDM2 inhibitor with a modified induction therapy treatment resulted in disease remission [13]. These results informed the design of an ongoing clinical trial (NCT04190550) testing the combination of an MDM2 inhibitor and induction therapy.

## 8. A Role for MTF2 in Breast Cancer

An important role of MTF2 regulation of the p53-induced apoptosis has also been established in breast cancer. Notably, MTF2 was expressed lowly in breast cancer tissue samples at different clinical stages, with a decreasing trend towards the higher stages of the disease, suggesting its role in cancer initiation and progression [17]. Moreover, the MCF-7 breast cancer cell line encoding wild-type TP53 showed lower MTF2 levels compared with MDA-MB-231 and HCC1937 cells that encode mutated TP53. Indeed, MTF2 was revealed to impair MDM2-mediated ubiquitination of p53 by sequestering MDM2 in the nucleolus, thereby stabilising p53 by preventing its MDM2-mediated degradation. Of note, though PHF1 was located primarily in the nucleus, MTF2 and PHF19 resided largely in the nucleolus. The biological implication of this finding is unclear. By performing a combination of wound healing, apoptosis, cell migration, and proliferation analyses, the authors demonstrated that overexpression of MTF2 was sufficient to inhibit invasion and migration of MCF-7 cells through inhibiting EMT, as mediated by E-cadherin and Vimentin [17]. This is consistent with mouse xenograft studies, which demonstrated that MTF2 overexpression significantly inhibits MCF-7 tumour growth in vivo [17]. HCC1937 cells, which lack intact TP53, resisted the pro-apoptotic impact of MTF2, suggesting a critical role for p53 mediating the effect of MTF2 on the aggressiveness of breast cancer. Specifically, MTF2-overexpressing cells showed elevated activation of p53 and its target genes (BAX and NOXA). Moreover, p21, a cyclin-dependent kinase inhibitor, was increased under such conditions, pointing to the role of MTF2 in regulating the cell cycle in breast cancer.

More recently, a similar association between low MTF2 expression and increased resistance to chemotherapeutics has also been reported in basal-like breast cancer cells (BLBC) [16]. A substantial proportion of BLBC patients rapidly develop resistance against cytotoxic therapies, experience high recurrence rates and have a dismal prognosis among breast cancers despite initial response to treatment [95]. Additionally, using RNA-seq and ChIP-seq, the PRC2 complex was found to promote drug resistance in breast cancer cells derived from the WAP-T mammary carcinoma mouse [16]. In fact, this group revealed that the decreased expression levels of Mtf2 and core PRC2 subunits resulted in breast cancer cells surviving cytotoxic treatment. Here, they demonstrated that reduced activity of PRC2 led to the de-repression of Nfat1c, which is involved in epithelial–mesenchymal transition (EMT) and resistance to cytotoxic treatments. Moreover, they also showed that inhibiting EZH2 activity by either small-molecule or genetic perturbation upregulates Nfatc1, leading to a refractory phenotype in breast cancer cells. Upregulation of Nfatc1 coincides with the loss of PRC2 activity and is directly implicated in the transcriptional changes in chemotherapy resistance. Notably, a pronounced epigenetic switch from the repressive H3K27me3 to the activating H3K27ac mark at regulatory regions of PRC2 target genes was revealed, confirming that chromatin structure is altered in BLBC chemoresistance. These observations show that PRC2 maintains low Nfatc1 expression levels and thereby represses aggressiveness and therapy resistance in BLBC [16]. However, athough MTF2 expression status is tracked with that of core PRC2 subunits, its underlying functional role and molecular mechanisms require further exploration. 

## 9. Oncogenic Activity of MTF2

In contrast to the role of MTF2 as a tumour suppressor in AML and breast cancer, MTF2 demonstrates oncogenic activity in other malignancies [18,19,20]. For example, in the blood cancer multiple myeloma, MTF2 and PHF19 were found to be upregulated compared to plasma cells from healthy individuals and were associated with high-risk disease [19,96]. Moreover, a forward genetics screen identified MTF2 and WDR26 as drivers of poor survival rates in multiple myeloma. Loss of either WDR26 or MTF2 by genetic ablation significantly impeded the growth of myeloma cells in vitro and in vivo via mouse xenograft assays, extending the survival of the xenografted mice [19]. Further investigation is needed to unravel the oncogenic networks involving MTF2, PHF19, and WDR26 in myeloma, as this could reveal novel opportunities for targeted molecular treatments and prevention strategies.

Retinoblastoma is an ocular tumour with poor prognosis that results from loss-of-function mutations in the tumour suppressor RB, accounting for 3% of childhood cancers [97]. Recently, Meng et al. [18] identified a potential role for MTF2 in retinoblastoma progression [18]. Specifically, they found that MTF2 is overexpressed in retinoblastoma. Using both gain- and loss-of-function perturbations in two retinoblastoma cell lines, they demonstrated that MTF2 is a target of the lncRNA colon cancer-associated transcript 1 (CCAT1)/miR-218-5p regulatory axis. CCAT1 competitive binding to miR-218-5p repressed the activity of miR-218-5p, including miR-218-5p-mediated repression of MTF2. Thus, overexpression of CCAT1 led to increased MTF2 abundance with concomitant elevated EMT, cell migration, and invasion of retinoblastoma [18]. Notably, lncRNA CCAT1 has been shown to induce proliferation and invasion mediated via sequestering miR-218-5p in several other malignancies including gallbladder [98], laryngeal squamous cell carcinoma [99], triple-negative breast cancer [100], and non-small-cell lung cancer [101], suggesting its widespread implication across cancers. However, whether *MTF2* is inhibited by miR-218-5p in other cancers besides retinoblastoma remains to be explored.

MTF2 has also been associated with the development and progression of glioblastoma, an aggressive brain tumour [102]. In this study, immunohistochemistry results from human glioma samples corroborated the results from the TCGA data analysis showing elevated MTF2 expression in glioblastoma multiforme (GBM) and lower-grade glioma. To test the role of MTF2 functionally, Wang et al. [102] overexpressed MTF2 in the human glioblastoma cell line U87 with adenoviral vector. U87 cells overexpressing MTF2 displayed higher proliferation. In contrast, down-regulating MTF2 in U251 cells (a human glioblastoma astrocytoma cell line) led to a reduced number of colonies, lower proportion of cells in the S phase of the cell cycle and higher apoptosis, indicating that deficiency of MTF2 halts the high proliferation phenotype in glioblastoma cells. Consistently, cells overexpressing MTF2 displayed higher levels of H3K27me3 as well as H3K4me2, with a decrease in H3K9me2. Of note, overexpression of MTF2 led to the enhanced expressions of the core component proteins of PRC2, such as EZH2 and EED, with a concomitant decrease in SUZ12. This observation indicates the multidimensional role of MTF2, such that it not only mediates PRC2 gene targeting, but it may also impact expression/stability of the core subunits [102]. Additionally, inhibition of EZH2 by small molecules nullified the cell growth-promoting property of MTF2, consistent with the oncogenic effects of MTF2 being PRC2-dependent.

MTF2 has also been reported to induce the progression and metastasis of hepatocellular carcinoma (HCC) [20]. In an analysis of HCC tissues from 43 afflicted patients, mRNA levels of *MTF2* were significantly higher in 81% of samples compared to that of normal liver cells. Consistent with transcript abundance, MTF2 protein abundance was enriched in malignant tissues. Moreover, patients with elevated MTF2 suffered from shorter overall and disease-free survival. Both overexpression and downregulation of MTF2 in several HCC cell lines (HepG2 and YY-8103 cells) confirmed the involvement of MTF2 in modulating cell growth and invasiveness, which was further validated in a xenograft mouse model. Moreover, MTF2 overexpression in HepG2 and YY-8103 cells was consistent with the aggressive nature of HCC. Collectively, these findings indicate the oncogenic property of MTF2 in hepatocellular carcinoma [20].

## 10. Bioinformatic Analyses of Public Databases

**Figure 3:**
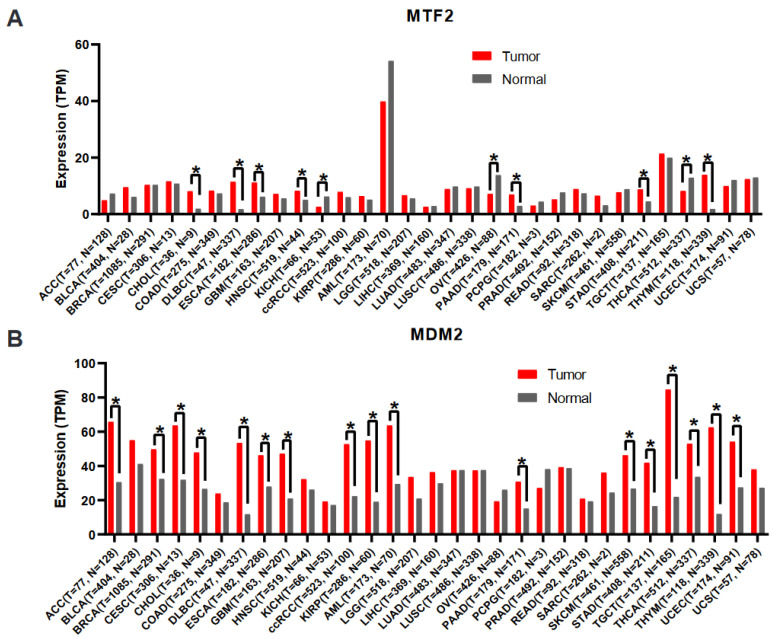
**MTF2 and MDM2 transcript expression levels in normal versus tumour tissues.** (**A**) *MTF2* transcript and (**B**) *MDM2* transcript levels are graphed across 31 tumours in comparison to their normal tissue counterparts. Both MTF2 and MDM2 display oncogenic activity in several cancer types. Low expression of *MTF2* is associated with elevated *MDM2* expression in ACC, AML, and THCA. ACC (adrenocortical carcinoma); BLCA (bladder urothelial carcinoma); BRCA (breast invasive carcinoma); CESC (cervical squamous cell carcinoma and endocervical adenocarcinoma); CHOL (cholangiocarcinoma); COAD (colon adenocarcinoma); DLBC (lymphoid neoplasm diffuse large B-cell lymphoma); ESCA (esophageal carcinoma); GBM (glioblastoma multiforme); HNSC (head and neck squamous cell carcinoma); KICH (kidney chromophobe); ccRCC (clear cell renal cell carcinoma); KIRP (kidney renal papillary cell carcinoma); AML (acute myeloid leukaemia); LGG (brain lower-grade glioma); LIHC (liver hepatocellular carcinoma); LUAD (lung adenocarcinoma); LUSC (lung squamous cell carcinoma); MESO (mesothelioma); OV (ovarian serous cystadenocarcinoma); PAAD (pancreatic adenocarcinoma); PCPG (pheochromocytoma and paraganglioma); PRAD (prostate adenocarcinoma); READ (rectum adenocarcinoma); SARC (sarcoma); SKCM (skin cutaneous melanoma); STAD (stomach adenocarcinoma); TGCT (testicular germ cell tumours); THCA (thyroid carcinoma); THYM (thymoma); UCEC (uterine corpus endometrial carcinoma); UCS (uterine carcinosarcoma). mRNA expression levels were obtained from TCGA data from the GEPIA2 (Gene Expression Profiling Interactive Analysis) web server [103]. Differentially expressed genes were determined based on Log2FoldChange = 0.58 and (*) q-value < 0.05. The bar plot represents the median expression of shown tumour type or normal tissue.

To determine whether MTF2 may play a critical role in the initiation, progression, diagnosis, prognosis, or chemotherapeutic resistance of additional cancers, we conducted an unbiased bioinformatic analysis of publicly available data and searched for patterns that may provide insight into what dictates whether MTF2 functions as a tumour suppressor or oncogene. To begin, we first analysed the mRNA expression levels of *MTF2* in various cancers using TCGA data from the GEPIA2 (Gene Expression Profiling Interactive Analysis) web server [103]. The resulting Figure 3 illustrates the median *MTF2* transcript values between tumour and normal tissue of the same organ. This analysis should identify cancers in which altered *MTF2* expression is common. As shown in Figure 3A, adrenocortical carcinoma, chromophobe renal cell carcinoma, AML, ovarian serous cystadenocarcinoma, adrenal gland cancers pheochromacytoma and paragngioma, prostate adenocarcinoma, and thyroid carcinoma show at least a 25% reduction in *MTF2* transcript levels compared to their normal counterpart tissue. In contrast, bladder urothelial carcinoma, cholangiocarcinoma, diffuse large B-cell lymphoma, esophageal carcinoma, GBM, head and neck squamous cell carcinoma, clear cell renal cell carcinoma, pancreatic adenocarcinoma, sarcoma, stomach adenocarcinoma, and thymoma show at least a 25% increase in *MTF2* expression compared to normal tissue.

These correlative data should not be over-interpreted. Mechanistic experiments that manipulate MTF2 expression are required to determine in which tissues MTF2 functions as a tumour suppressor, oncogene, or neither. However, since MTF2 regulates MDM2 expression and/or activity in AML and breast cancer [13,17], we integrated MDM2 transcript levels into our analysis. Notably, in addition to AML, adrenocortical carcinoma and thyroid carcinoma demonstrate decreased *MTF2* levels and concomitant elevated *MDM2* expression (Figure 3B), suggesting that loss of MTF2 in adrenocortical carcinoma and thyroid carcinoma could impact disease progression and therapeutic response. In fact, it has been reported that adrenocortical carcinoma cell lines are responsive to small-molecule MDM2 inhibition in vitro [104], and in preclinical studies, combined MDM2 and MEK inhibition prolongs survival of thyroid carcinoma xenograft mice [105]. In contrast, transcript levels of both MTF2 and MDM2 are elevated in diffuse large B-cell lymphoma and esophageal carcinoma, whereas both MTF2 and MDM2 transcripts are down-regulated in malignant ovarian cancer (serous cystadenocarcinoma). These data are consistent with the complex regulation of MDM2 expression transcriptionally and post-transcriptionally [106,107].

**Figure 4:**
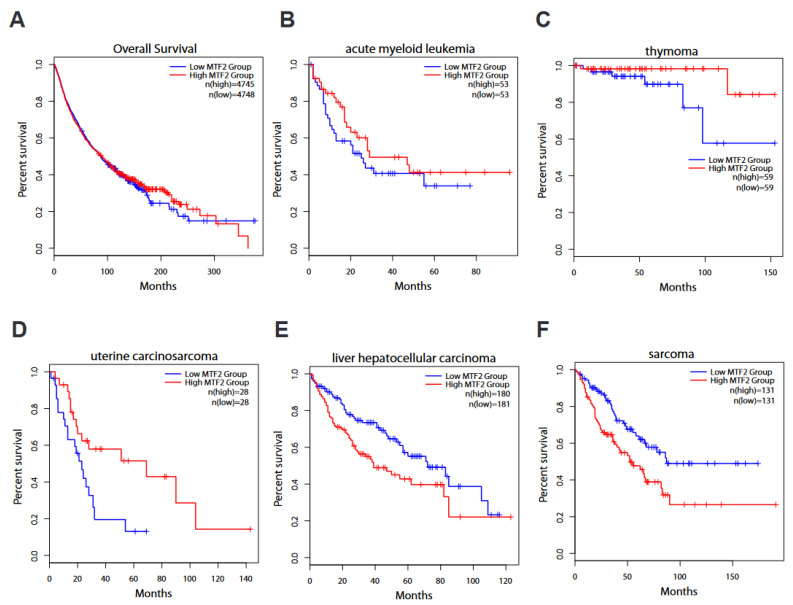
**Kaplan–Meier survival rate based on MTF2 expression level.** (**A**) Overall survival of patients with low MTF2 level (n = 4748) tends to be dire compared to their counterparts with high MTF2 (Logrank p = 0.71, n = 4745). (**B**) Elevated MTF2 expression in patients with acute myeloid leukaemia (AML) is concomitant with promising survival, although it does not reach a significant cut-off value (Logrank p = 0.26, n = 53). (**C**) Thymoma (THYM) patients with elevated MTF2 have a substantially favourable survival rate (Logrank p = 0.044, n = 59). (**D**) Uterine carcinosarcoma (UCS) patients survive longer when MTF2 is higher (Logrank p = 0.018, n = 28). (**E**) Liver hepatocellular carcinoma (LIHC) with elevated MTF2 results in dire survival rate (Logrank p = 0.0026, n (high) = 180, n (low) = 181). (**F**) Sarcoma (SARC)-inflicted patients with higher MTF2 levels demonstrate dismal prognosis (Logrank p = 0.002, n = 131). TCGA data from GEPIA2 web server were used for survival analyses.

We next performed Kaplan–Meier survival analysis of TCGA data from the GEPIA2 web server. Due to the lack of control tissues in the TCGA datasets, patients were categorised as “high MTF2” if the RNA-seq MTF2 reads were greater than the mean MTF2 reads of the cancer dataset versus “low MTF2”, in which the RNA-seq transcript levels were below the mean MTF2 expression. In addition to the arbitrary definition of “high” versus “low” patient cohorts, other caveats of these TCGA datasets are that the genomic landscape represents only a snapshot in time, and both treated and untreated patients are included in the analyses. Even with these limitations in mind, it is interesting to note that Kaplan–Meier analyses of all available GEPIA2 data revealed that cancers with low MTF2 mRNA levels trend towards poor overall survival rates (Figure 4A). Confirming our previous findings [13], survival of “MTF2-low” AML patients is reduced in this independent TCGA dataset (Figure 4B). Strikingly, although thymoma tumours generally express higher levels of MTF2 than non-disease thymic tissue (Figure 3A), “MTF2-low” thymoma patients have lower survival rates (Figure 4C). Additionally, uterine carcinosarcoma patients within the “MTF2-low” group also have lower survival rates (Figure 4D).

Consistent with the reports discussed above that MTF2 has oncogenic activity in HCC [20], hepatocellular carcinoma patients with higher levels of *MTF2* expression showed reduced survival rates compared to “MTF2-low” patients (Figure 4E). The strong correlation between high MTF2 levels and poor overall survival in sarcoma patients (Figure 4F) indicates that mechanistic studies of MTF2 in sarcoma are warranted. In summary, the Kaplan–Meier analyses confirm published AML and hepatocellular carcinoma studies while underscoring potential prognostic or mechanistic roles of MTF2 in thymoma, uterine carcinosarcoma, and sarcoma. We advocate for studies to be performed in these cancers to determine whether MTF2 plays a role in disease pathogenesis or may be useful as a biomarker.

**Figure 5:**
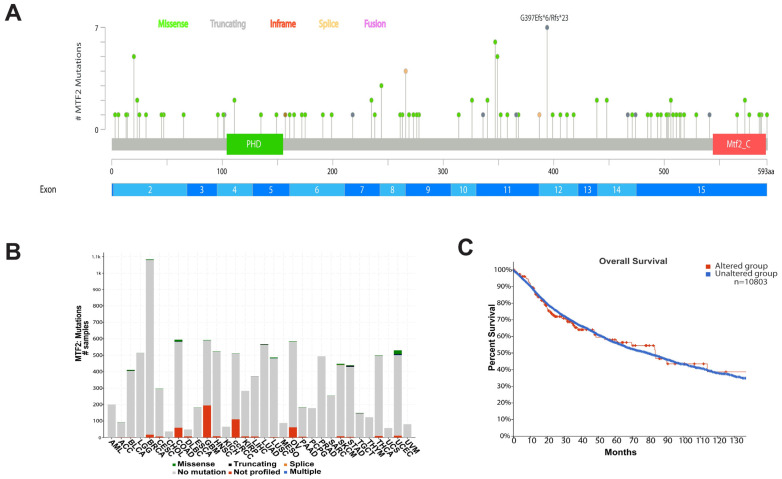
**MTF2 mutational analysis in cancer**. (**A**) Identification of all detected mutations across the MTF2 gene. (**B**) Various types of mutations (missense, truncating, splice, multiple) are shown across all 32 cancer types. In all cancerous tissues, the MTF2 gene has not been affected by any mutations with the exception of UCEC (uterine corpus endometrial carcinoma), which shows several mutations such as missense types. (C) Overall survival of patients with mutated versus nonmutated MTF2 gene is similar (Logrank p = 0.757, n = 1080).

We next analysed the mutational status of *MTF2* to determine its potential as a driver mutation of various cancer types using cBioportal data. In cBioPortal, *TP53* stands out as one of the most frequently mutated tumour suppressors, with a mutation rate of 36%. Conversely, PIK3CA ranks as one of the prominently mutated oncogenes, with a mutation rate of 17%. Consistent with the rarity of *MTF2* mutations in AML, the incidence of *MTF2* mutations is very low across cancer types, with a recorded mutation rate of 1.5% observed among 10,953 patients encompassing 32 different cancer types (Figure 5A,B). The vast majority of mutations that have been detected are missense mutations distributed throughout the gene body (Figure 5A,B). Among all 32 analysed cancer types, uterine corpus endometrial carcinoma and colon adenocarcinoma had the highest mutation rates (missense mutation). Consistent with the low mutation frequency, the cBioportal data revealed no known driver mutations in the MTF2 gene. Furthermore, *MTF2* mutation status did not significantly affect the survival rate of patients (Figure 5C).

**Figure 6:**
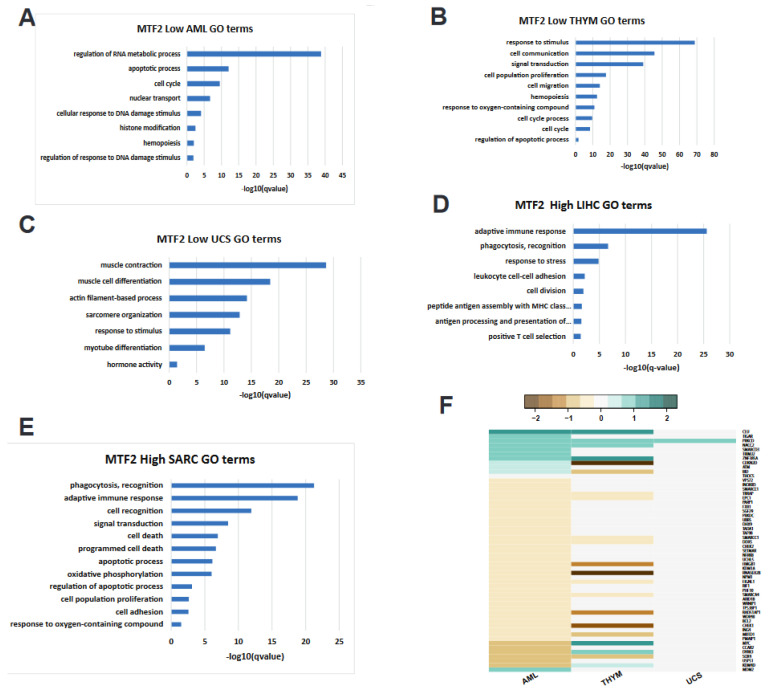
**GO enrichment terms of divergent MTF2 expression levels in cancer**. (**A**) Low abundance of MTF2 in AML dysregulates several pathways, including regulation of RNA metabolic process and DNA damage response (DDR). (**B**) Thymoma-inflicted patients with reduced MTF2 display dysregulated cell cycle and apoptosis, to name a few effects. (**C**) Affected functional pathways in UCS patients with reduced MTF2 are shown; no biological processes were affected similarly with either AML- or thymoma-diagnosed patients. (**D**,**E**) Elevated *MTF2* transcript levels in LIHC and SARC are associated with enrichment of several pathways. Both tumours respond distinctly to increased MTF2, such that LIHC immune system-related pathways are enriched; in SARC, apoptosis and cell proliferation processes are affected. (**F**) Heatmap of Log2FoldChange exhibiting the list of differentially expressed genes in low-expressing MTF2 AML, THYM, and UCS samples. Most affected genes in MTF2-low AML are involved in DNA damage response process. PRKCD (which functions in nonhomologous end joining DNA repair) is the only commonly differentially affected gene. The Gene Ontology (GO) analysis was performed using the g:Profiler web server, and the cut-off value was set at adj. p-value < 0.05.

We sought to determine whether unbiased pathway analysis across cancers could provide insight into tumour suppressor versus oncogenic functions of MTF2. We performed Gene Ontology (GO) enrichment analysis on a TCGA cohort of cancers with low (AML, thymoma, and uterine carcinosarcoma) and high (hepatocellular carcinoma and sarcoma) *MTF2* expression levels. In the AML samples with low MTF2, we identified genes dysregulated in processes such as the cell cycle, regulation of the RNA metabolic process, nuclear transport, the apoptotic process, histone modification, and the DNA damage response processes (DDR) (Figure 6A), in agreement with the biological processes we detected in our local MTF2-deficient AML cohort [13]. In thymoma patients with low MTF2, we also observed cell cycle and regulation of the apoptotic process, confirming low similarity with MTF2-low AML samples (Figure 6B). In contrast, analysis of dysregulated pathways in MTF2-low uterine carcinosarcoma patients showed no significant similarity with either AML or thymoma patients (Figure 6C). Comparably, in cancer types with high *MTF2* transcript levels, hepatocellular carcinoma and sarcoma also had low similarity with each other (Figure 6D,E). Furthermore, MTF2-low AML samples showed the most differentially expressed DDR process-related genes. Thymoma revealed intermediate differential expression, and uterine carcinosarcoma showed protein kinase C delta (PRKCD) as the only detected differentially expressed gene, consistent with the GO term analysis. Notably, PRKCD showed increased expression across MTF2-low AML, thymoma, and uterine carcinosarcoma. PRKCD belongs to a member of the calcium-independent novel PKCs and exerts a broad spectrum of biological functions such as growth, apoptosis, mitochondrial function, and tumorigenicity [108]. Importantly, PRKCD is involved in DNA damage response [109]. Together, these data point to differential *MTF2* mRNA levels exerting effects on different underlying biological processes that may be fundamental to the various cancer types, tissues, and microenvironments and identifies PRKCD as a potential pathogenic target of MTF2 regulation.

## 11. Future Directions 

PcG proteins are essential regulators of cell fate decisions that contribute to physiological and pathological processes. Their divergent, often contradictory roles in cancer render them an attractive avenue of extensive enquiry. The loss of PRC2 activity has been associated with the development of various haematological malignancies, including AML and myelodysplastic syndrome (MDS), but also with the prevention of MDS progressing to AML, highlighting a dual role for PcG proteins in these diseases, as outlined in this review [52]. Specifically, accessory subunits associated with PRC2 have emerged as key cofactors in the organisation and spatiotemporal catalytic activities of the complex. A simplistic model of how PRC2 can be a tumour suppressor in one type of cancer and an oncogene in another would be that the differential use of accessory proteins would drive these divergent roles. Our review of the literature and bioinformatic analyses of publicly available data refute this model, as MTF2 functions as a tumour suppressor or an oncogene, depending on the tissue context.

There are many ambiguities regarding PRC2 complex constituents in general that may contribute to its components’ contrasting roles in cancer. For example, it is far from clear how MTF2 competes with other accessory subunits for binding PRC2 core complex and what determines its spatial- and temporal-specific roles. Notably, the potential upstream modulators or PRC2-independent functions of MTF2 in epigenetic and onco-epigenetic contexts have not been defined. Furthermore, the question of whether MTF2 plays any compensatory roles for other accessory subunits has not been adequately explored. The exact number of different PRC2 variants formed in any given cancer type and functional crosstalk between those different variants is unknown. PRC2.1 complex has exhibited high-affinity chromatin-binding behaviour compared to PRC2.2, thereby favouring PRC2.1 in biological systems that require robust transcriptional silencing of PcG-regulated genes, such as stem cells [110]. Thus, detecting the underlying pathways regulating the formation of one subcomplex over another would provide contextual regulatory properties of subunits such as MTF2. Inducible expression and degradation systems such as auxin-inducible degron technology to regulate MTF2 expression in a host of cancer cell lines would be valuable tools to reveal the functions of MTF2 in a context-specific fashion.

Finally, there is a paucity of information on posttranslational modifications of MTF2 and their functional impact. For instance, it has been shown that EZH2 switches from a repressor to a co-activator upon phosphorylation of S21, mediated directly or indirectly by the PI3K/AKT pathway for critical transcription factors, including the androgen receptors [24]. Determining whether MTF2 function is modified by specific posttranslational modifications could provide critical insight into the divergent roles of MTF2 in cancer. Hence, mass spectrometry approaches designed to identify posttranslational modifications in MTF2 in various cancer cell lines should be considered. Furthermore, immunoprecipitation-based proteomics analyses could reveal tissue-specific co-factors that moderate the MTF2 function in cancer.

## 12. Materials and Methods

### 12.1. Gene Expression Analysis

We performed MTF2 and MDM2 gene expression analysis across 33 tumours from The Cancer Genome Atlas (TCGA) (https://www.cancer.gov/ccg/research/genome-sequencing/tcga: accessed on 22 February 2023) and the GTEx dataset and paired normal tissues using the GEPIA2 web server (http://gepia2.cancer-pku.cn/: accessed on 10 February 2023). Differentially expressed genes were identified with the cut-off of Log2FoldChange of 0.58 and q-value < 0.05. The bar plot represents the median expression of the shown tumour type or normal tissue.

### 12.2. Survival Analysis 

The Kaplan–Meier method with the log-rank test was used to calculate the overall survival of the TCGA cancer patients using the GEPIA2 web server for *MTF2* expression and cBioportal (https://www.cbioportal.org/: accessed on 19 January 2023) for *MTF2* mutation survival. Hazard ratio (HR) and 95% confidence intervals (CIs) were analysed by the Cox proportional hazards regression model. Group cut-off was set at median values. A log-rank p-value < 0.05 was taken as statistically significant.

### 12.3. Mutational Analysis

The prevalence and location of the mutations were analysed using the cBioportal database. The specific dataset used was the TCGA, which contained 10,803 samples.

### 12.4. RNA-Seq Analysis 

Metadata and RNA-seq raw count data of corresponding survival groups of AML, thymoma, uterine carcinosarcoma, hepatocellular carcinoma, and sarcoma were obtained from TCGA. Once the count data were normalised, we identified differentially expressed genes between groups at a cut-off value of adj. p-value < 0.05. This was done using DESeq2 in the R platform [111].

### 12.5. GO Term Analysis

After identifying DEGs, we performed functional enrichment analysis to identify enriched biological processes. The Gene Ontology (GO) analysis was performed using the g:Profiler web server (https://biit.cs.ut.ee/gprofiler/: accessed on 28 February 2023); the cut-off value was set at adj. p-value < 0.05. The Log2FoldChange heatmap was drawn using the heatmap2 tool in Galaxy [112].

## Data Availability

The data used in this article were obtained from cBioportal (https://www.cbioportal.org/ accessed on 19 January 2023), The Cancer Genome Atlas (TCGA) (https://www.cancer.gov/ccg/research/genome-sequencing/tcga accessed on 22 February 2023), and the GEPIA2 web server (http://gepia2.cancer-pku.cn/ accessed on 10 February 2023).
